# Surgical Management of a Completely Avulsed Adductor Longus Muscle in a Professional Equestrian Rider

**DOI:** 10.1155/2014/828314

**Published:** 2014-02-19

**Authors:** Conal Quah, Andrew Cottam, James Hutchinson

**Affiliations:** ^1^Royal Derby Hospital, Uttoxeter Road, Derby DE22 3NE, UK; ^2^ST6 Trauma and Orthopaedic Surgery, East Midlands North Rotation, UK; ^3^Queen Alexandra Hospital, Southwick Hill Road, Cosham, Portsmouth PO6 3LY, UK

## Abstract

Avulsion injuries of the adductor longus muscle tendon are rare and a challenge to manage especially in athletes. There has been little published literature on the outcome of conservative and operative treatment for these injuries. We report the first case of an acute adductor longus avulsion injury which was surgically repaired in a professional equestrian rider. Return to full preinjury function was achieved at 3 months with surgical repair using 3 suture anchors.

## 1. Introduction

Groin injuries are common and account for approximately 5–18% of all athletic injuries with a higher reported incidence in kicking sports [1]. These injuries are often severe and disabling, requiring a lengthy period of recovery. Avulsion injuries of the adductor longus muscle tendon are rare and a challenge to manage especially in athletes. As the exact mechanism of injury is often unclear, the approach to management is difficult and results can be unpredictable. There has been little published literature on the outcome of conservative and operative treatment for these injuries.

Most injuries occur proximally at the musculotendinous junction although there have been several reports of injuries occurring at the proximal and distal parts of the adductor longus muscle [1–9]. Tears at the origin are infrequent, with only a few cases reported in the literature [1–5]. Nearly all of these cases were associated with kicking sports and were reattached surgically.

We report a case of an acute adductor longus avulsion injury in a professional equestrian rider. To the best of our knowledge, this is the first case report that has demonstrated the successful outcome of bone anchor repair with objective muscle power testing postoperatively.

## 2. Case Summary 

A 43-year-old professional equestrian presented to the Accident and Emergency Department complaining of acute groin and lower abdominal pain after horse riding. She describes the pain coming on suddenly whilst jumping a hedge. It was associated with a “snapping sensation” in her left groin as she gripped her horse tightly with her knees while standing in the stirrups.

**Figure 1:**
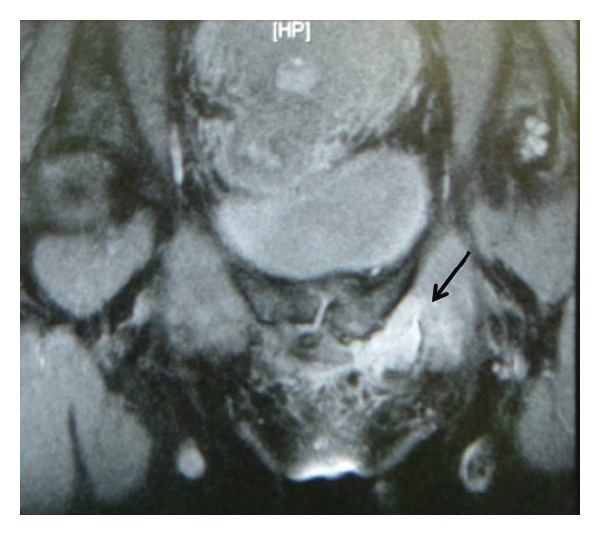
High signal changes in left superior pubic rami indicating bone oedema secondary to avulsion of the adductor longus tendon from its origin.

On examination, there was significant swelling with extensive ecchymosis from her inner thigh extending up to her pubic and left inguinal area. She was tender to palpation over the superomedial aspect of her thigh with a palpable gap at the origin of the adductor longus tendon. Adductor muscle power was weak (Medical Research Council grade 2). Magnetic Resonance Imaging (MRI) of the pelvis demonstrated an avulsion of the adductor longus tendon to a distance of 1.2 cm from its origin on the pubis (Figure 1). We felt that reattachment of the avulsed muscle would give her the best and quickest chance of returning back to her horse riding.

## 3. Surgical Technique

**Figure 2:**
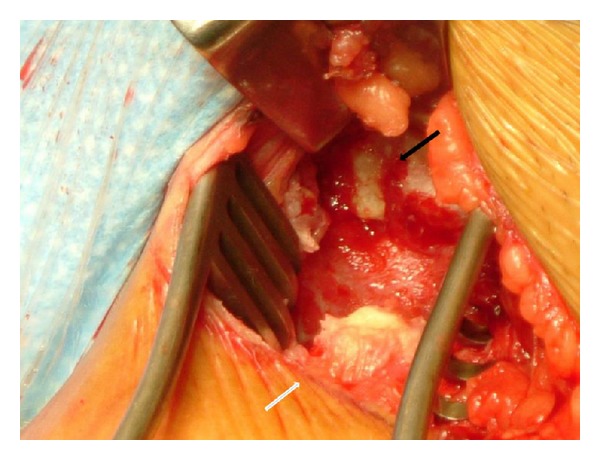
A bare area (black arrow) on the pubic symphysis where the avulsed adductor longus tendon (white arrow) was previously attached.

The patient was positioned supine with her left hip flexed and abducted. A lower groin skin crease incision was made with careful blunt dissection down to the pubic symphysis. A bare area on the pubic symphysis where the avulsed tendon was previously attached was located along the proximal end of the tendon 2 cm distally (Figure 2).

**Figure 3:**
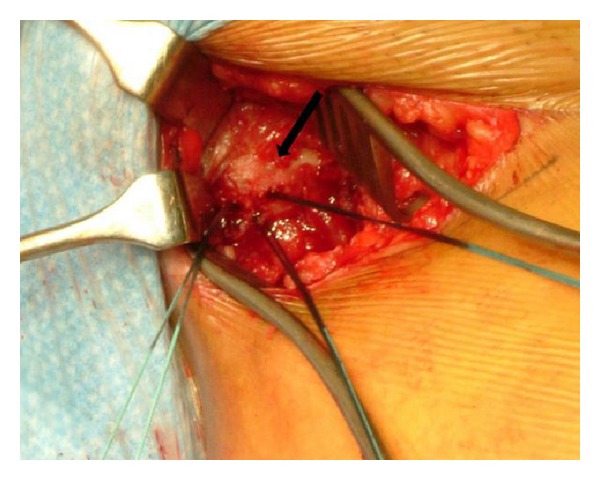
The avulsed adductor longus tendon end was reattached to the pubis (black arrow) with 3 suture bone anchors.

The bony and avulsed end of the adductor tendon was freshened with a blade and reattached to the pubis with 3 suture anchors. They were inserted into the bone with good hold and sutured to the tendon using a modified Kessler-type technique (Figure 3).

The tendon sheath was repaired and the wound was thoroughly washed out and closed in layers. Postoperatively the patient wore an abduction brace for 6 weeks and had enoxaparin as thromboprophylaxis.

## 4. Rehabilitation

At 2 weeks postoperatively, the wound had healed well, with no signs of infection. At this point the patient was fully weight bearing without any pain. She undertook no strenuous activities, including horse riding for 3 months. At 3 months she had recovered full power in her adductor muscle and was asymptomatic. Muscle power was tested and compared to the right side using a Kin-Com Dynamometer with software version 5.30HS3. Both right and left side muscles were tested in a range of positions from 18 degrees of abduction to 14 degrees of adduction. The results showed that the left leg had 102% of the power of the right leg for eccentric contraction and 161% of the power of the right leg for concentric contraction; that is, the left side was at full power 3 months after repair. She returned to her previous level of activities at this point with no residual symptoms.

## 5. Discussion

There is limited literature available on the management of proximal adductor longus tendon injuries. Distal ruptures seem to be more common with some recommending acute repair [4]. Interestingly most of these cases described a mechanism of injury of wide hip abduction and extension with some internal rotation, a very similar position that our patient was in whilst riding. Proximal ruptures tend to occur more commonly in kicking sports such as American football, soccer, and rugby [1, 2, 8].

Patients with proximal adductor longus rupture usually present with an acutely painful groin often describing the injury as a sharp “snapping” pain. Examination will reveal swelling and tenderness of the inner thigh and potentially a palpable gap at the origin of the adductor longus tendon [1–9]. Often extensive haematoma of the groin, thigh, and external genitalia will be present. In the absence of high velocity trauma (where plain radiographs may be useful to exclude fracture) an MRI scan is the investigation of choice.

In a few of the previously documented cases of proximal adductor longus tendon rupture, the technique used for surgical repair was slightly different to the method we described [3, 5]. In one case, the osseous avulsion of the adductor tendon was reattached to the periosteum of the pubis using titanium corkscrews and Fiberwire number 2 (Arthrex, Naples, FL, USA) [5]. The patient returned to full activity without pain in 8 weeks following treatment. In another report of 2 cases, nonabsorbable sutures and bone anchors were used using a “locking whip stitch” technique [3]. Both patients returned to full activity without pain at 3 months after surgery. Surgical excision of the ruptured muscle has been described as an option for treatment; however, return to full function was reported only at 18 months [4].

In our case 3 mitek suture anchors were used to repair the avulsed adductor longus tendon. At 3-month followup, the patient was back to normal level of physical activities. Objective muscle testing of her repaired adductor longus muscle revealed full return of power. This might not have been the case if her injury was managed conservatively. In previous studies which have looked at muscle power of the adductor longus muscle after tenotomy for chronic groin pain, weakness has been noted to be a particular problem. Akermark and Johansson found that only 63% of the athletes in their series were able to resume their previous level of competition [7]. Since it is generally accepted that tenotomy of the adductor longus muscle results in decreased muscle strength and level of activity, it follows that repair of acute ruptures avoids these problems. However, in a more recent study, Schlegel et al. compared nonoperative to operative repair [8]. They found that, although both groups returned to play in the National Football League, the mean time to return to play was more than twice as long in the surgical group. One of the limitations of this study was that it did not objectively test muscle strength after treatment. Given the retrospective nature of this study's design, there was also no standardized nonoperative rehabilitation program.

## 6. Conclusion

Proximal rupture of the adductor longus tendon is a rare injury, particularly avulsion injures at the pubic bone. This type of injury can take a lengthy time to recover if managed conservatively. We believe that surgical repair using 3 mitek suture anchors is an option worth considering especially in active individuals.
